# Role of Deep Eutectic Solvent Precursors as Hydrotropes: Unveiling Synergism/Antagonism for Enhanced Kraft Lignin Dissolution

**DOI:** 10.1021/acssuschemeng.4c02529

**Published:** 2024-05-31

**Authors:** Filipe H. B. Sosa, Dinis O. Abranches, André M. da Costa Lopes, Mariana C. da Costa, João A. P. Coutinho

**Affiliations:** 1 CICECO, Aveiro Institute of Materials, Department of Chemistry, University of Aveiro, 3810-193 Aveiro, Portugal; 2 CECOLAB—Collaborative Laboratory Towards Circular Economy, R. Nossa Senhora da Conceição, 3405-155 Oliveira do Hospital, Portugal; 3 School of Chemical Engineering (FEQ), University of Campinas (UNICAMP), 13083-852, Campinas, São Paulo, Brazil

**Keywords:** Kraft lignin, Deep eutectic solvents, Solubility, Hydrotropy, Aqueous solutions

## Abstract

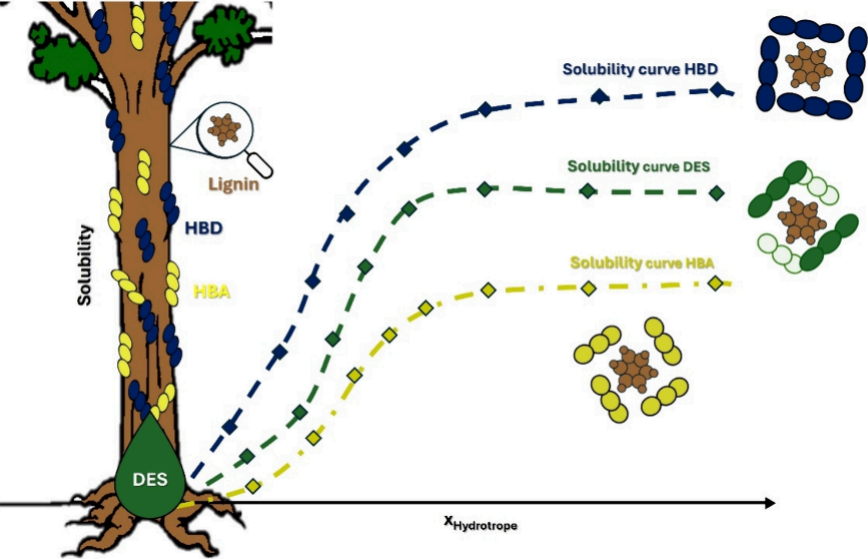

Lignin holds significant potential as a feedstock for generating valuable aromatic compounds, fuels, and functional materials. However, achieving this potential requires the development of effective dissolution methods. Previous works have demonstrated the remarkable capability of hydrotropes to enhance the aqueous solubility of lignin, an amphiphilic macromolecule. Notably, deep eutectic solvents (DESs) have exhibited hydrotropic behavior, significantly increasing the aqueous solubility of hydrophobic solutes, making them attractive options for lignin dissolution. This study aimed at exploring the influence of hydrogen bond acceptors (HBAs) and hydrogen bond donors (HBDs) on the performance of DESs as hydrotropes for lignin dissolution, while possible dissolution mechanisms in different water/DES compositions were discussed. The capacity of six alcohols (glycerol, ethylene glycol, 1,3-propanediol, 1,4-butanediol, 1,5-pentanediol, and 1,6-hexanediol) and cholinium chloride to enhance the solubility of Kraft lignin in aqueous media was investigated. A correlation between solubility enhancement and the alkyl chain length of the alcohol was observed. This was rationalized upon the competition between hydrotrope–hydrotrope and solute–hydrotrope aggregates with the latter being maximized for 1,4-butanediol. Interestingly, the hydrotropic effect of DESs on lignin solubility is well represented by the independent sum of the dissolving contributions from the corresponding HBAs and HBDs in the diluted region. Conversely, in the concentrated region, the solubility of lignin for a certain hydrotrope concentration was always found to be higher for the pure hydrotropes rather than their combined HBA/HBD counterparts.

## Abstract 2

This study is on the potential of deep eutectic solvents as hydrotropes for lignin dissolution, exploring the impact of hydrogen bond acceptors and hydrogen bond donors on their performance.

## 1 Introduction

The growing demand for the consumption of nonrenewable resources has driven the search for renewable and sustainable sources. In this sense, lignocellulosic materials have attracted attention from the scientific community due to their advantages such as availability, low cost, and biodegradability.^1,2^ Lignocellulosic materials are composed of cellulose (40–60%), hemicellulose (10–25%), and lignin (25–35%).^3^ Lignin is a complex macromolecule with an aromatic character and can be used to produce value-added compounds.^4−7^ However, its natural amphiphilicity, complexity, high chemical stability, and low solubility in conventional solvents, including water, hamper the processing and utilization of lignin.^8,9^ Consequently, lignin is often used in low added value applications, despite being a large reserve of carbon with high potential as an aromatic commodity.^10,11^

Reliable, cheap, and nontoxic solvents, applicable to different types of lignins, are highly desired for lignin dissolution. Recently, ionic liquids (ILs) and deep eutectic solvents (DESs) have emerged as promising alternatives for processing biomass fractions.^12−15^ However, there are still some disadvantages inherent to the use of ILs at industrial scale.^16^ For this reason, DESs have been highlighted as a good alternative. DESs are mixtures of hydrogen bond donors (HBDs) and hydrogen bond acceptors (HBAs) whose temperature at the eutectic point is lower than that of an ideal mixture, presenting negative deviations from thermodynamic ideality.^17,18^

A few works have explored lignin solubility in DESs.^19−21^ Francisco et al.^13^ reported the solubility of lignin and cellulose in DESs formed by combinations of cholinium chloride ([Ch]Cl) and carboxylic acids or amino acids. They concluded that all DESs displayed low to negligible cellulose solubility but high lignin solubility. Lynam et al.^22^ observed this same behavior in solubility for lignin, cellulose, and isolated hemicelluloses. Liu et al.^23^ proposed new combinations for the formation of DESs, using lactic acid or *N*-methylthiourea as HBDs and four different quaternary ammonium salts as HBAs. The authors found that these DESs can efficiently dissolve different types of lignin including calcined lignin, enzymatic hydrolysis lignin, sodium lignosulfonate, and Organosolv lignin.

Additionally, some authors have demonstrated the correlation between Kamlet–Taft parameters and lignin solubility in DES and ionic liquids (ILs). The results show that DES/ILs with higher values of β and β–α (net basicity) are expected to efficiently dissolve cellulose, have greater net basicity, and are favorable for lignin dissolution. Moreover, the solubility of xylan was linearly correlated with the β value.^24,25^

The effect of water on the dissolution of lignin is equally important and Kumar et al.^26^ studied the impact of adding water during the extraction of lignin. The authors concluded that the addition of a small amount of water during the delignification of rice straw significantly improved the extraction of lignin, demonstrating that the addition of water contributes to the dissolution of lignin.^27^ Soares et al.^21^ identified that DES aqueous solutions allowed an increase in the solubility of Kraft lignin higher than 1180-fold, and the results suggested the presence of a hydrotropic mechanism behind the solubility of lignin in aqueous solutions of DESs.

Hydrotropes can considerably increase the ability of water to dissolve hydrophobic solutes.^21,28−30^ Besides, depending on the hydrotrope concentration in the system, adding a small amount of water can lead to the precipitation of the solute, facilitating its recovery if necessary.^29,31,32^ This is very interesting for DESs, since they can exhibit amphiphilicity and act as a hydrotope. Their properties, such as polarity and viscosity, can be adjusted by changing the relative concentrations of their components.

In general, the hydrotropic effect has been described as the formation of solute–hydrotrope complexes through π–π interactions.^33−35^ Still, the formation of water-mediated contacts between the apolar regions of the hydrotrope and the solute has gained strength with new evidence.^34,36^ This theory proposes that the hydrotropic effect occurs due to an accumulation of hydrotropic molecules around the solute. In this case, the water mediates the aggregation of the apolar fractions of the hydrotropes around the solute, decreasing the global nonpolar surface area in contact with water and increasing its hydrogen bonding ability.^20,37^

Another point relevant to be evaluated is the molar ratio of DESs (HBD/HBA), since it is known to play an important role in the physicochemical properties of the resulting eutectic mixture.^38^ By changing the HBA/HBD molar ratio, the melting temperature of the solvent is also altered, leading to a solid, a liquid, or a combination of both at room temperature.^39^ Therefore, it is fundamental to understand the effect of HBD/HBA molar ratios on lignin solubility. Soares et al.^21^ studied the solubilities of monomeric lignin model compounds and technical lignins (Organosolv and Kraft) in several DES aqueous solutions. According to their results, the molar ratio significantly impacts the solubility of lignin monomers. Lignin solubility increased with higher HBD molar ratios.

Additionally, the water concentration in the DES is another important parameter in lignin dissolution. In literature, different studies have reported that adding water or ethanol had a negative impact on the capacity of ChCl-based DESs to dissolve lignin, since interactions between solvent and electronegative Cl^–^ are favored. On the opposite, the addition of water significantly enhanced mass transfer and activated reaction sites in proline-based DESs, thereby improving or maintaining lignin solubilities at high water concentrations.^40,41^

In our previous study,^42^ Kraft lignin solubility was enhanced by increasing the HBD molar ratio, while adding water provided an opposite behavior. However, this negative impact of water was less pronounced for DESs composed of long alkyl chain HBDs. In other words, larger HBD alkyl chain lengths allowed maintaining lignin solubility for higher water contents. Therefore, it can be inferred that these DESs, and especially HBDs, self-organize around lignin macromolecules in the presence of water. This behavior is crucial for dissolving lignin and maintaining its solubility in aqueous media. Furthermore, the sigmoidal profile of the obtained lignin solubility curves, which were fitted using the model developed by Shimizu and Matubayasi,^43^ suggests a cooperative intermolecular interaction involving hydrotropic molecules that participate in the dissolution process, as reported by Balasubramanian et al.^44^

Encouraged by the previous works mentioned above, this study aims at further spanning the understanding of lignin solubility in aqueous solutions of DESs, particularly by inspecting the individual contributions of HBAs and HBDs to the hydrotropic mechanism of lignin dissolution. Additionally, most of the literature data exploring the solubility of organic molecules in DESs are based on fixed HBA/HBD stoichiometries. Therefore, the methodological approach addressed in this work provides more in-depth research upon the effect of HBA/HBD molar ratio on lignin dissolution in DESs. In this framework, the solubility of Kraft lignin in aqueous solutions of alcohols and [Ch]Cl and their combinations at different molar ratios were investigated. Alcohol-based DESs were selected by taking into account that they were reported to be nonderivatizing solvents of lignin, thus being able to dissolve lignin without modifying its chemical structure.^42^

## 2 Materials and Methods

### 2.1 Chemicals

**Figure 1:**
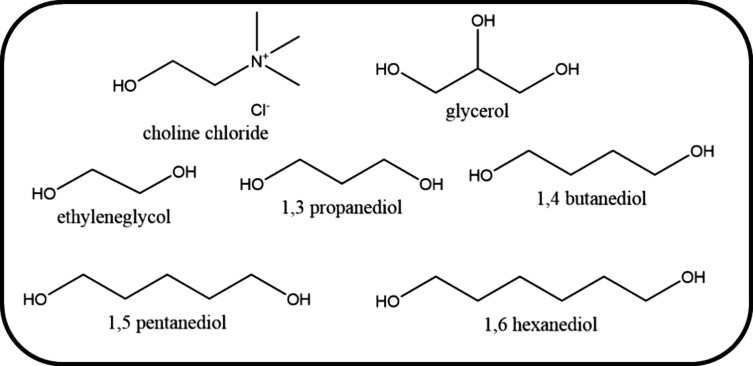
Chemical structures of [Ch]Cl and HBDs investigated in this study.

Six combinations of [Ch]Cl and alcohols were used to prepare DESs and to evaluate the effects of the HBDs structure on the solubility of Kraft lignin. All chemicals used in this work are summarized in Table 1, and their structures are represented in Figure 1. Kraft lignin from *Eucalyptus urograndis* was directly supplied by Suzano S.A. (Brazil). The isolation process was addressed by adding carbon dioxide and sulfuric acid (H_2_SO_4_) to the industrial black liquor, followed by several washing steps. The resulting Kraft lignin presents 95% purity.

**Table 1:** Name, CAS Number, Molecular Weight (Mw), Mass Fraction Purity, and Supplier for the DES Components Investigated in This Work

compound name (abbrev)	CAS number	Mw, g mol^–1^	purity,^a^ wt %	supplier
Cholinium chloride ([Ch]Cl)	67-48-1	139.62	98.0	Acros Organics
Glycerol (GLY)	56-81-5	92.09	99.0	Acros Organics
Ethylene glycol (EGLY)	107-21-1	62.07	99.0	Fluka
Propane-1,3-diol (PROP)	504-63-2	76.10	98.0	Sigma
Butane-1,4-diol (BUT)	110-63-4	90.12	99.0	Alfa Aesar
Pentane-1,5-diol (PENT)	111-29-5	104.15	97.0	Alfa Aesar
Hexane-1,6-diol (HEXA)	629-11-8	118.16	97.0	Aldrich
Kraft lignin	8068-05-1	2500.00	95.0	Suzano S.A.

a As reported by the supplier.

### 2.2 DES Preparation

The mixtures of [Ch]Cl and HBDs at specific molar ratios were placed in sealed glass vials with a stir bar and heated in a paraffin bath at 333.15 ± 0.01 K with constant stirring for about 2 h or until the formation of a clear and colorless liquid.^12^ After preparation, the water content was measured using a Metrohm 831 Karl Fischer coulometer, and determined values were taken into account when preparing DES aqueous solutions.

### 2.3 Lignin Solubility

The lignin solubility tests were conducted as described by Sosa et al.^42^ Excess lignin was added to glass vials containing aqueous solutions of DESs or aqueous solutions of HBDs or HBAs. The flasks were sealed and placed in an aluminum block holder on a heating plate (LBX Instruments H03D series) with magnetic stirring and temperature control (PT100). The samples were kept under stirring at 313.15 K until reaching saturation (24 h) and then were filtered using PTFE filters (0.45 μm pore size) separating the solubilized lignin from the insoluble lignin. The resulting liquid phase was diluted with dimethyl sulfoxide (99.98%, Fischer Scientific, USA), and the concentration of dissolved lignin was determined by UV spectroscopy (Synergy HTX Multi-Mode Reader from BioTek Instruments) at a wavelength of 280 nm. All results were obtained in triplicate.

### 2.4 Modeling

The solubility data were adjusted using the cooperative model of hydrotropy developed by Shimizu and Matubayasi, which is derived from statistical thermodynamics.^36^ This model was developed to describe the usual sigmoidal solubility curves found in hydrotropy while also capturing information about the interactions between solute and hydrotropic molecules. The model developed by Shimizu and Matubayasi^36^ can be described in a linearized form as
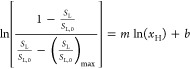
1where *x*_H_ = *x*_HBD_ or *x*_H_ = *x*_ChCl_ or *x*_H_ = *x*_DES_ = *x*_HBD_ + *x*_ChCl_ and where *S*_L_ is the solubility of lignin in the system (mol L^–1^), *S*_L,0_ is the solubility of lignin in water, and *x*_H_ is the molar fraction of the hydrotrope, in this case being alcohol, [Ch]Cl, or the sum of the two in the case of a DES. The term *S*_L_/*S*_L,0_ represents the relative solubility of lignin in the solution with respect to that in pure water. The parameters *m* and *b* represent the molecular interactions between the solute and hydrotrope. More specifically, *m* represents the number of hydrotrope molecules in the vicinity of the solute. After determining the parameters *m* and *b*, it is possible to determine the model curve fitted to the experimental data.

## 3 Results

### 3.1 Lignin Solubility Curves in HBD Aqueous Solutions

**Figure 2:**
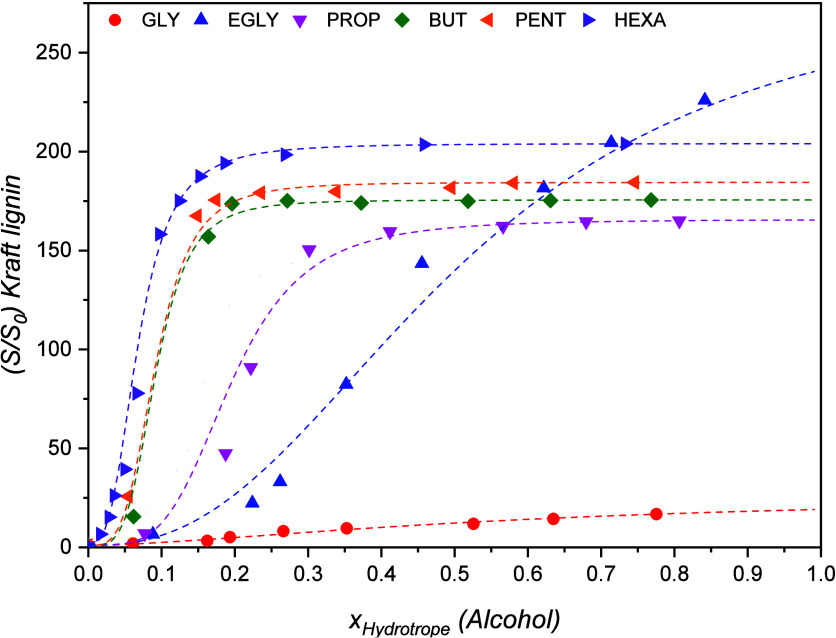
Solubility data for Kraft lignin in aqueous solutions of glycerol (GLY), ethylene glycol (EGLY), 1,3-propanediol (PROP), 1,4-butanediol (BUT), 1,5-pentanediol (PENT), and 1,6-hexanediol (HEXA) at 313.15 K, fitted using the cooperative hydrotropy model (- - -). *S*_0_ = 0.146 wt %.

To understand the influence of HBDs and HBAs on the dissolution of lignin, we conducted an initial assessment of the solubility curves of Kraft lignin in aqueous solutions containing individual HBD alcohols (namely, glycerol, ethylene glycol, 1,3-propanediol, 1,4-butanediol, 1,5-pentanediol, or 1,6-hexanediol), or [Ch]Cl, at a temperature of 313.15 K, as depicted in Figure 2 or Figure S1 ([Ch]Cl), respectively. Note that *S* and *S*_0_ represent the solubility (mol L^–1^) of Kraft lignin in aqueous solutions of alcohols and pure water, respectively. The linearized curves of Kraft lignin in aqueous solutions are presented in the Supporting Information (Figure S1 and Figure S2).

Based on the lignin solubility curves obtained (Figure 2), it can be assumed that the dispersive interactions facilitated by the alkyl chains of alcohols play a significant role in dissolving lignin and sustaining its solubility in aqueous environments. Moreover, the sigmoidal shape of the solubility curves and the excellent fit achieved using the Shimizu and Matubayasi hydrotropy model suggest that the solubility of Kraft lignin in these systems (alcohols and [Ch]Cl) is primarily driven by a hydrotropic mechanism.^31,36^ In other words, the alcohols and [Ch]Cl have a tendency to self-organize around lignin macromolecules in the presence of water, thereby maintaining their solubility. This self-organization is amplified as the carbon chain length of the hydrogen bond donor (HBD) increases, leading to stronger dispersive forces between the alkyl chains.

Figure 2 shows that increasing the size of the alkyl chain of the alcohol improved the solubility of lignin, particularly within the diluted range (at low hydrotrope concentrations), as evidenced by the Setschenow constants (Table S1), with hydrotrope performance being ranked as GLY < EGLY < PROP < BUT < PENT < HEXA. Note how the aqueous solubility of lignin can be increased up to 200-fold in this diluted region using 1,6-hexanediol as the hydrotrope. However, despite having a larger alkyl chain, the solubility enhancement provided by glycerol is lower than that provided by ethylene glycol. The presence of an extra hydroxyl group in glycerol enhances its hydrogen bonding capacity, which in turn increases its preference to form hydrogen bonding networks with water, hampering its aggregation around lignin.^21,31,45,46^

Despite the clear trend between the hydrotropic capacity and alkyl chain length identified in the previous paragraphs, Figure 2 reveals an interesting phenomenon for ethylene glycol. Outside the diluted hydrotropic region where water ceases to be the main solvent, ethylene glycol proves to be the most promising system for lignin dissolution (*S*/*S*_0_ = 226), since its lignin solubility curve does not follow the typical sigmoidal shape observed for the remaining hydrotropes studied. This behavior is not related to hydrotropy, which occurs when water represents the majority of the solvation environment of a solute and can thus mediate hydrophobic interactions. Instead, ethylene glycol’s behavior may be attributed to the synergistic effect between its compact size and the presence of two hydroxyl groups, providing stronger ability in establishing hydrogen bonds with lignin compared to other alcohols.^47^ Therefore, a mixed-polarity and low molar mass organic solvent seems to be ideal for lignin dissolution in neat solvents. Regardless, even glycerol, the least effective hydrotrope studied in this work, exhibited an almost 20-fold increase in the rate of dissolution in water, much higher than the results found for traditional solvents.^31^

In addition, the solubility results discussed above also indicate that water content is a crucial factor influencing alcohol’s ability to dissolve lignin. That is, the longer their alkyl chain length (or apolar volume), the lower is the amount of hydrotrope necessary to reach the maximum solubility enhancement of lignin, which then remains constant upon further hydrotrope addition. This also means that systems with hydrotropes composed of longer alkyl chains are more resilient to water addition. For example, HEXA maintains its maximal ability to enhance the solubility of lignin in an extensive water mole fraction window, from a neat hydrotrope up to a water mole fraction of 0.8.

**Figure 3:**
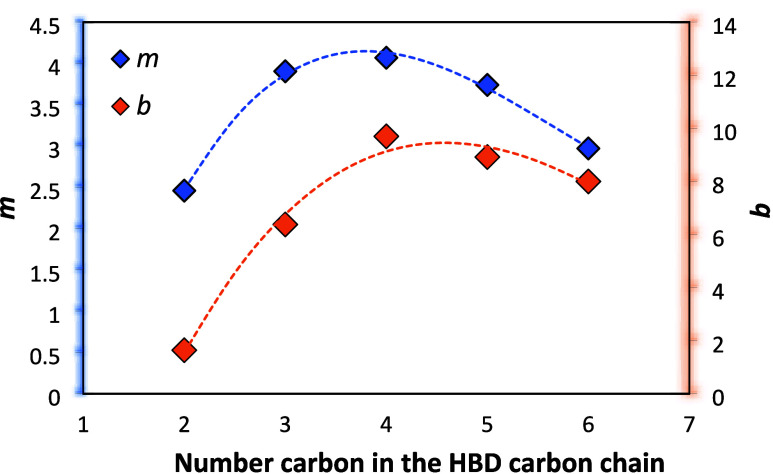
Number of carbons in the HBD carbon chain versus “*m*”(blue tilted square) and “*b*” (orange tilted square).

The “*m*” parameter in the cooperative hydrotropy model distinctly reaches a maximum based on the number of carbons in the alkyl chain of the HBD (Figure 3, Table S2). This parameter, which is independent of the hydrotrope concentration, is related to the average number of hydrotropes aggregated around a given solute molecule. As shown in Figure 3, *m* and *b* reaches its maximum value for 1,4-butanediol.

The hydrophobic effect is the driving force of hydrotropy and governs the extent of water-mediated contacts between the apolar moieties of both the solute and hydrotrope molecules. This is the reason behind the increase in lignin solubility with increasing hydrotrope alkyl chain length. As discussed above, larger hydrotrope apolar volumes lead to more extensive water-mediated hydrotrope–solute interactions. However, increasing the apolar volume of the hydrotrope also enhances hydrotrope–hydrotrope aggregation through precisely the same hydrophobic mechanism. Thus, the maximum value of *m* mentioned above can be interpreted by considering competing hydrotrope–solute and hydrotrope–hydrotrope interactions. In other words, increasing the apolar volume of the hydrotrope simultaneously heightens solute–hydrotrope and hydrotrope–hydrotrope aggregation. The maximal driving force for solute–hydrotrope aggregation is expected to be found when the apolar volume of the hydrotrope is roughly the same as that of the solute, decreasing thereafter.^46^ As indicated in Figure 3, this maximum (here quantified through *m*) occurs at 1,4-butanediol.

It is worth noting that the extent of solute–hydrotrope aggregation is not necessarily correlated with hydrotropy efficiency.^46^ In other words, despite displaying the largest *m* value and thus the largest extent of solute–hydrotrope aggregation, 1,4-butanediol still exhibits a lower ability to enhance the solubility of lignin when compared against, for example, the larger 1,6-hexanediol. Although this hydrotrope is statistically less aggregated around lignin, hexanediol–lignin contacts are still more extensive in terms of the apolar area covered than their butanediol–lignin counterparts. This precludes the disruption of hydrogen bonding in a larger number of water molecules, which would otherwise be in contact with the apolar surface of lignin, leading to a superior solubility enhancement of lignin.

Overall, these findings demonstrate the applicability of the Shimizu and Matubayasi^36^ hydrotropy model in describing lignin solubility within these systems. This approach enables the correlation, interpretation, and understanding of lignin solubility data, a task rendered challenging by the proximity of the melting point of lignin with its decomposition point, impeding the precise determination of its melting point using the solid–liquid equilibrium (SLE) method.

### 3.2 Lignin Solubility Curves in DES Aqueous Solutions: Influence of HBA and HBD

#### 3.2.1 Diluted Region

The interpretation of how the combination of HBAs and HBDs affects lignin solubility is made difficult by the various potential interactions among water, hydrotropes (HBAs, HBDs, or DESs), and the solute. Each of these components plays distinct roles, either promoting or hindering the solute solubility. Although this complexity is more prone at higher hydrotrope concentrations, in diluted regions (with low hydrotrope concentration, *x*_H_ < 0.3), the contribution of hydrotrope–hydrotrope and solute–solute interactions to dissolution has been found to be significantly reduced.^46^ Therefore, investigating the diluted region should offer a more comprehensive understanding of the hydrotrope–solute interaction.

In order to understand the effect of HBA and HBD on the dissolution of Kraft lignin in alcohol-based DES aqueous solutions, the solubility data obtained in the previous work for DESs^42^ and this work for neat HBDs were compared. The purpose of this comparison relied on assessing whether the solubility enhancement of lignin in DESs can be described as simply the sum of the contributions of each DES component ([Ch]Cl and each HBD studied in the previous section) or if synergistic/antagonistic effects are present. The assessment can be performed by using the cooperative hydrotropy model that was fitted to the experimental data through the following approach:
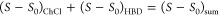
2
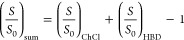
3where (*S* – *S*_0_)_ChCl_ and (*S* – *S*_0_)_HBD_ represent the independent contributions of ChCl (HBA) and HBD to lignin dissolution, while (*S* – *S*_0_)_sum_ represents the sum of these independent contributions. Thus, synergism/antagonism can be inferred when  differs from that observed experimentally for DESs.

**Figure 4:**
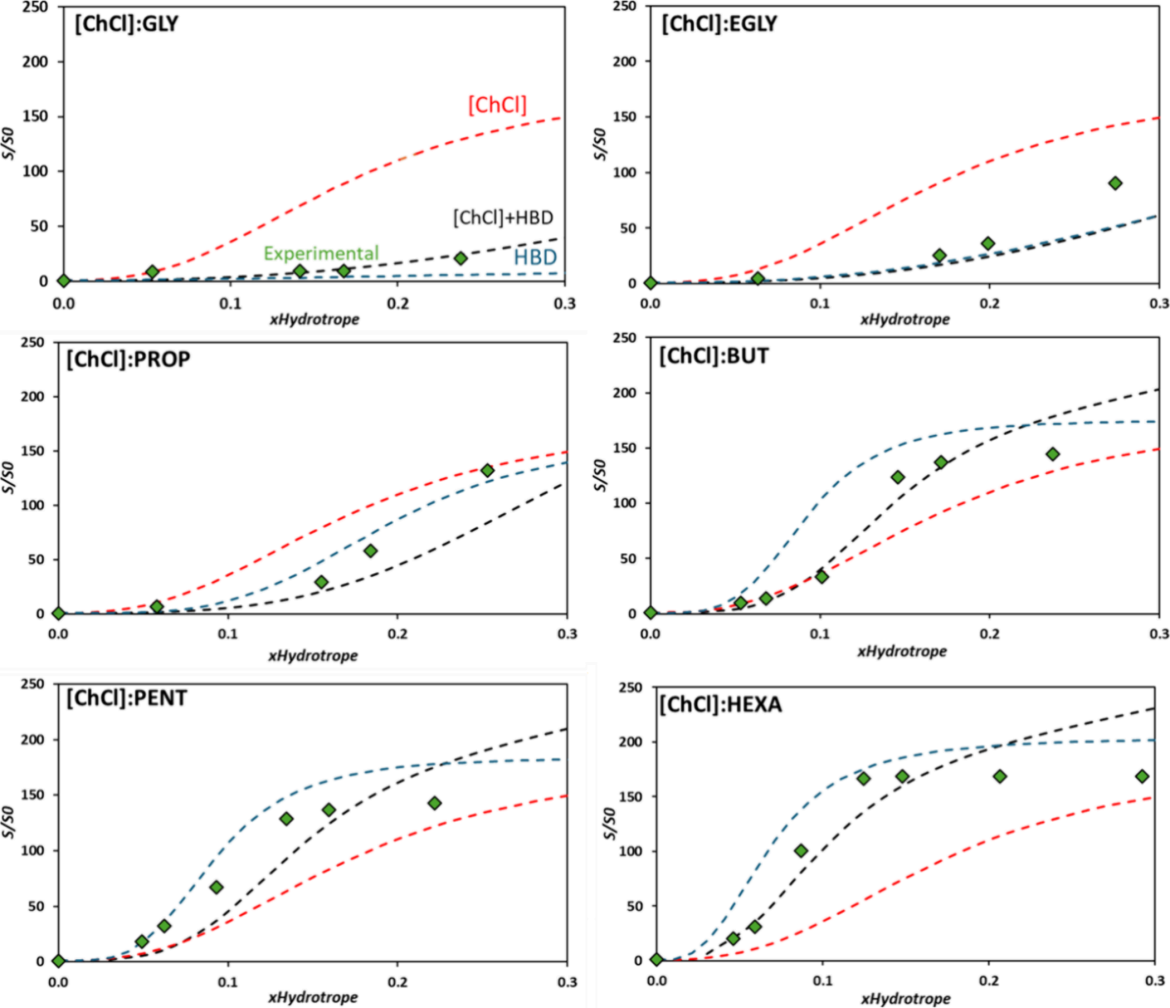
Experimental solubility data for Kraft lignin in aqueous solutions of [ChCl]:GLY, [ChCl]:EGLY, [ChCl]:PROP, [ChCl]:BUT, [ChCl]:PENT, and [ChCl]:HEXA (1:2) at 313.15 K, fitted using the cooperative hydrotropy model for [ChCl] (red dashes), HBD (blue dashes), and sum of the independent contributions of [ChCl] and HBD (black dashes). *S*_0_ = 0.146 wt %. *x*_Hydrotrope_ = *x*_DES_ = *x*_HBD_ + *x*_ChCl_ or *x*_Hydrotrope_ = *x*_ChCl_ or *x*_Hydrotrope_ = *x*_HBD_.

Figure 4 shows the experimental data on the solubility of Kraft lignin (emphasis given to the diluted hydrotrope region) in various alcohol-based DES with a molar ratio of 1:2 (HBA:HBD) (green diamonds) and the fitted curves using the cooperative hydrotropy model for [Ch]Cl (red dashed lines). Additionally, it includes the fitted model using the cooperative hydrotropy model for each corresponding neat HBD (blue dashed lines) and the sum of the independent contributions of [Ch]Cl and the HBD (black dashed lines, eq 3). This contribution was determined using the fitted lignin solubility curves in the aqueous solutions of [Ch]Cl and alcohols (Figure 2, Figure S1, and Table S3).

The model that considers the effect of DESs on lignin solubility as the sum of independent contributions from HBA and HBD (black dashed lines) roughly reproduces the experimental data trends before the solubility plateaus, with small exceptions for ethylene glycol and propanediol. In those two systems, some sort of synergistic mechanism between [Ch]Cl and the HBD is observed, which makes the experimental lignin solubility higher than that predicted from the independent DES component contributions. On the other hand, discrepancies observed for ChCl:BUT, ChCl:PENT, and ChCl:HEXA when the experimental lignin solubility curves reach a plateau may be connected with the simplistic approach of eqs 2 and 3 and do not necessarily indicate antagonism between DES precursors, as will be explained in the following section.

Perhaps more relevant than the absence or presence of synergism is the fact that the use of DESs is counterproductive toward the enhancement of lignin solubility. This means that for all cases depicted in Figure 4, the solubility of lignin is always higher in systems containing a single hydrotrope rather than corresponding DES. For ChCl:GLY, ChCl:EGLY, and ChCl:PROP mixtures, [Ch]Cl provides a higher increase of lignin solubility than DES for any given mole fraction (red curve always above the experimental data points), while for ChCl:BUT, ChCl:PENT, and ChCl:HEXA mixtures, the HBD (BUT, PENT, HEX) always provides a higher solubility enhancement (the blue curve is always above the experimental data points).

There is a competition between HBD–HBA interactions and HBD/HBA–solute interactions in aqueous solutions of DESs. The HBA and HBD within the DES interact through hydrogen bonds, forming a specific structure that defines the properties of these solvents.^18^ This structure may reduce the availability of these components to effectively interact with lignin, thereby limiting the dissolution capacity of the DESs compared to its pure components.^48^ While pure components, i.e., HBD and HBA individually, may exhibit intrinsic affinity for lignin dissolution, their simultaneous presence in an aqueous solution can lead to a competition for these interactions.

This observation raises the consideration that the application of DES for lignin dissolution may not always be advantageous compared with the pure components, especially in aqueous solutions. On the other hand, depending on the application, the use of the mixture (DES) can exhibit a synergistic effect. da Costa Lopes et al.^49^ assessed the role of [Ch]Cl in the delignification of biomass containing DES with lactic acid and paratoluenosuofonic acid. The authors reported that the halide counterion increases the cleavage rate of β-O-4 and, consequently, enhances the biomass delignification rate. Yet, the synergistic effect goes beyond the dissolution properties of the solvent to the catalytic effect on the cleavage of lignin chemical bonds to enable lignin extraction from biomass.^50^

#### 3.2.2 Hydrotrope-Rich Region

In comparison to the diluted region discussed in the previous section, the hydrotrope-rich region exhibits a notable increase in the significance of hydrotrope–hydrotrope and solute–solute interactions in the process of dissolution. These interactions must not be overlooked, making the proposed equation (eq 3) inappropriate for this scenario.

**Figure 5:**
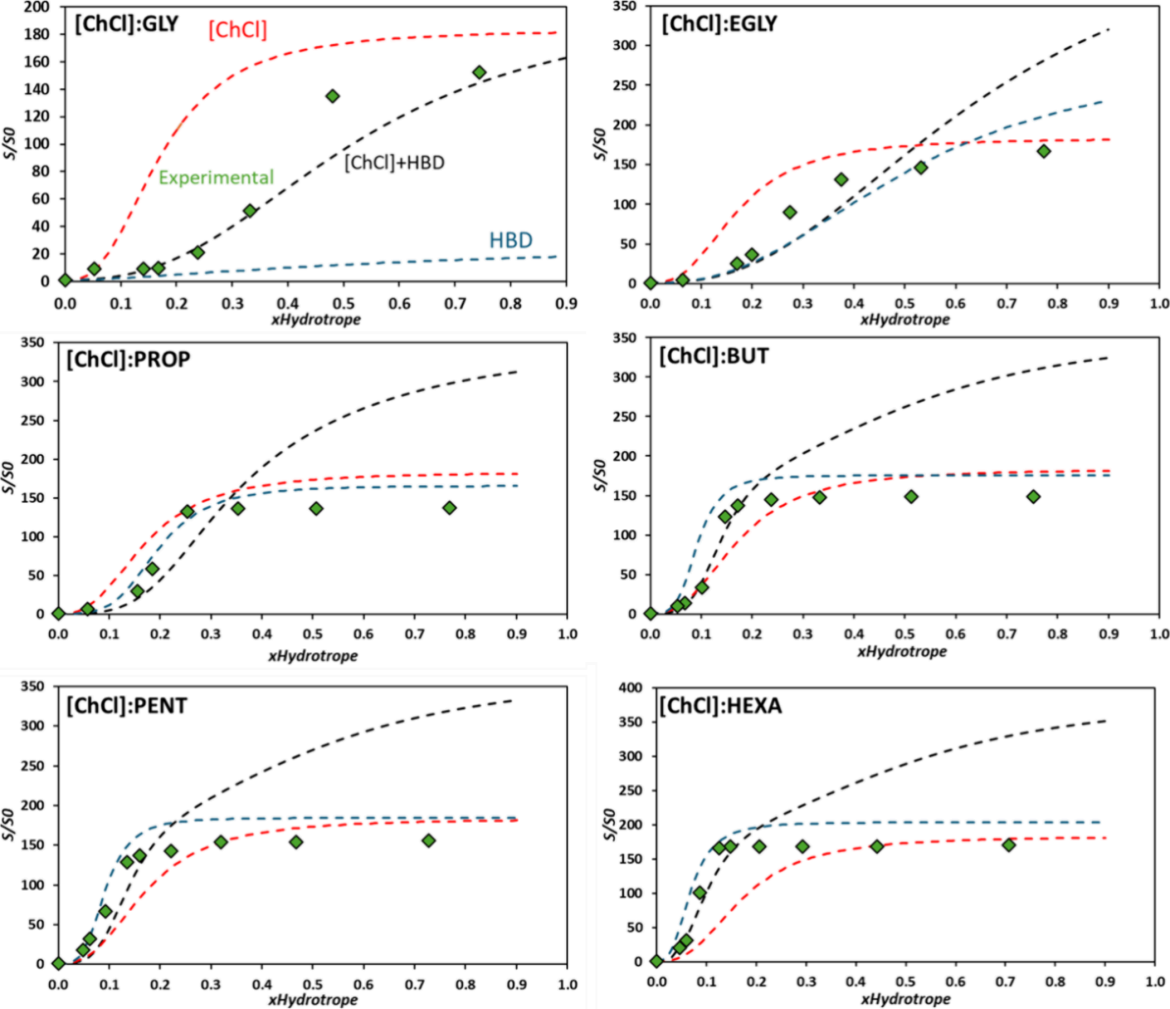
Experimental solubility data for Kraft lignin in aqueous solutions of [ChCl]:GLY, [ChCl]:EGLY, [ChCl]:PROP, [ChCl]:BUT, [ChCl]:PENT, and [ChCl]:HEXA (1:2) at 313.15 K, fitted using the cooperative hydrotropy model for [ChCl] (red dashes), HBD (blue dashes), and sum of the independent contributions of [ChCl] and HBD (black dashes). *S*_0_ = 0.146 wt %.

In this regard, the solubility of Kraft lignin was assessed across the entire experimentally available concentration range of the hydrotrope, covering from pure water to the pure hydrotrope or its aqueous solubility limit. Figure 5 presents the experimental data depicting the solubility of Kraft lignin in various alcohol-based DES, along with the fitted model utilizing the cooperative hydrotropy model for [Ch]Cl and HBD. Similar to the observations in the diluted phase, as the length of HBD carbon chain increases, lignin solubility is also enhanced in the nondiluted phase. In this region, the influence of water is minimal or negligible on the performance of these DES, resulting in a plateau in lignin solubility values.

Much like the results depicted in Figure 4, Figure 5 reveals that for all studied systems, the experimental lignin solubilities obtained are always below those that represent the cooperative hydrotropy model of one of the pure compounds. In other words, the solubility of lignin for a certain hydrotrope concentration will always be higher for the pure hydrotrope than for their combination (DES) at the same hydrotrope concentration.

Taking the behaviors of ChCl:PENT and ChCl:HEXA as examples, it is possible to note that the experimental solubility data are below that of the pure alcohol model curve (blue dashed line), indicating that in these cases the presence of [Ch]Cl is counterproductive for Kraft lignin dissolution. Therefore, the best solubility enhancement of lignin is obtained using neat 1,6-hexanediol rather than using its DES counterpart (with [Ch]Cl) (Figure 5). In the case of systems containing alcohols with shorter alkyl chains ([ChCl]:GLY, [ChCl]:EGLY, and [ChCl]:PROP), the same effect is observed; however, it is the alcohol that either does not assist or hinders the dissolution of lignin, while [Ch]Cl demonstrates the best hydrotropic behavior, as supported by the higher solubility values obtained for neat [Ch]Cl.

### 3.3 Effect of HBD/HBA Molar Ratio on Lignin Solubility

Considering that aqueous solutions of alcohols, particularly those with a higher carbon chain length (HEXA), exhibit considerable potential for lignin dissolution, while the formation of a corresponding DES with [Ch]Cl appears to be counterproductive, the subsequent step involved investigating the impact of the HBA:HBD molar ratio.

The HBD:HBA molar ratio plays an important role in the physical (viscosity and density) and chemical (ionicity, pH, and electrical conductivity) properties of DESs. By changing the HBD:HBA molar ratio, a liquid, a viscous liquid, a solid, or even a combination of both can be obtained.^48^ Therefore, it is essential to understand the effect of HBDs, HBAs, and their molar ratios on the solubility of Kraft lignin as well as to observe the existence of a possible synergistic effect between HBDs and HBAs.

**Figure 6:**
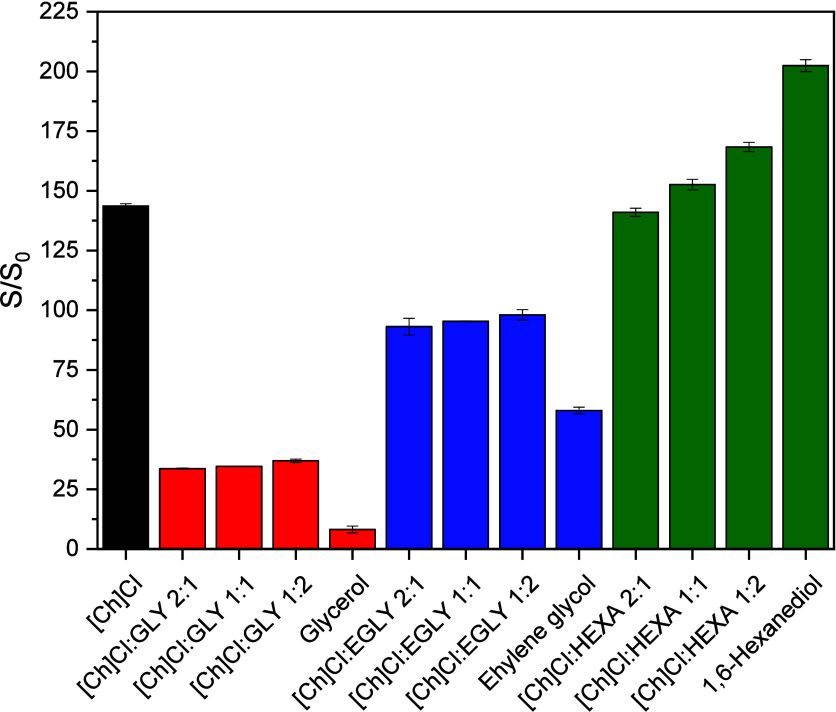
Influence of HBD molar ratio on the solubility enhancement of Kraft lignin in alcohol-based DES with 70 mol % water content at 313.15 K. *S*_0_ = 0.146 wt %.

For this purpose, 3 alcohol-based DESs ([Ch]Cl:GLY, [Ch]Cl:EGLY, [Ch]Cl:HEXA) with different molar proportions were studied in aqueous solutions with 70 mol % water content at 313.15 K (Figure 6). At this water content, the undiluted region of the system is represented, allowing for the evaluation of the hydrotrope–hydrotrope and solute–solute interactions in the process of lignin dissolution. According to the results shown in Figure 6, the molar ratio has a significant impact on improving Kraft lignin solubility, mainly in the HEXA solvent system.

For GLY and EGLY solvent systems at a 70 mol % water content, maximum dissolution was observed for aqueous solutions of [Ch]Cl (*S*/*S*_0_ = 143.6), while minimum dissolution was observed for pure alcohols, as detailed in the previous section. In this system, the use of DES (HBA + HBD) as a hydrotrope was found to be less effective for lignin dissolution compared to that of pure [Ch]Cl. Furthermore, for these solvent systems under these conditions, no significant effect of the molar ratio of HBA to HBD on the lignin solubility was observed.

In contrast to the results discussed in the previous paragraph, for solvent systems involving HEXA, where the dissolution potential of the pure alcohol exceeds that of [Ch]Cl, it was observed that the lignin solubility increased with the molar ratio of HEXA. The lignin solubility reached a maximum for the solution with pure HBD. These results suggest that no synergistic effect exists for this system.

**Figure 7:**
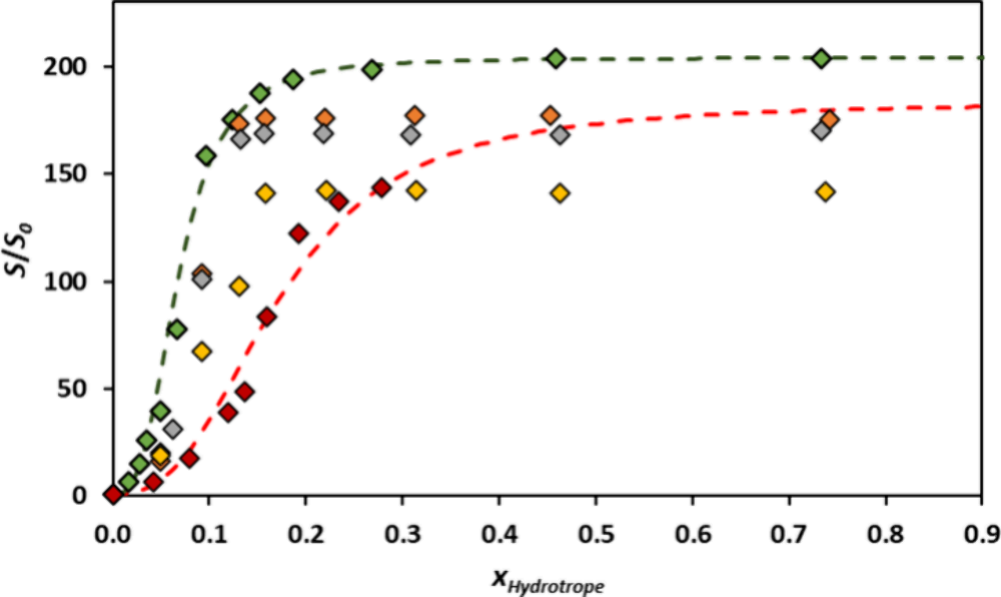
Experimental solubility data for Kraft lignin in aqueous solutions of [Ch]Cl (red tilted square), [Ch]Cl:HEXA (1:1) (yellow tilted square), [Ch]Cl:HEXA (1:2) (gray tilted square), [Ch]Cl:HEXA (1:3) (orange tilted square), and HEXA (green tilted square) at 313.15 K and fitted using the individual hydrotropy model for [Ch]Cl (orange dashes) and HEXA (green dashes). *S*_0_ = 0.146 wt %.

The impact of the molar ratio of HBA/HBD was also addressed in more detail for the [Ch]Cl:HEXA solvent system. Lignin solubility data using aqueous solutions at different molar ratios of [Ch]Cl and HEXA (pure [Ch]Cl, 1:1, 1:2, 1:3, and pure HEXA) were obtained (Figure 7). In this system, pure HEXA exhibited the highest lignin solubility values across the studied region, showing a plateau in the region with *x* > 0.2.

By increasing the amount of [Ch]Cl in DES, the lignin solubility decreased and achieved the lowest lignin solubility value for pure [Ch]Cl. Interestingly, with the lowest amount of [Ch]Cl in DES (1:3), a higher solubility was obtained. This can be advantageous depending on the system’s application, especially in biomass delignification processes with DES, where the role of chloride anion is important.^49^

## 4 Conclusions

The solubility of Kraft lignin was examined in aqueous solutions containing cholinium chloride, various alcohols, and their corresponding DESs, revealing their hydrotropic potential. Experimental data were analyzed by using the cooperative hydrotropy model. The results showcased the impressive hydrotropic ability of alcohols, significantly boosting the lignin solubility in aqueous solutions and surpassing the water performance by up to 200 times. Additionally, the study elucidated the impact of the HBA/HBD molar ratio and the distinct contributions of HBA and HBD in Kraft lignin dissolution.

In the diluted region, the hydrotropic effect of DESs is roughly represented as the sum of individual contributions from the corresponding HBAs and HBDs. On the other hand, in the concentrated region, a consistent observation revealed that the solubility of lignin at a specific hydrotrope concentration is consistently higher for pure hydrotropes compared to their combination within DES. Overall, this study underscores the significance of separately evaluating the application of HBA and HBDs for lignin dissolution, particularly in aqueous solutions, where the melting point of the mixtures is less pertinent. It is hoped that the results reported here will enable a greater understanding of the impact of the HBA/HBD ratio on the solubility of Kraft lignin (and that of other types of lignin) to contribute to the development of green lignin valorization.

## Supporting Information Available

Experimental solubility data and the fitting using the cooperative hydrotropy model for Kraft lignin (Figures S1 and S2); Setschenow constants for lignin in solvents (Table S1); list of parameters of the cooperative hydrotropy model for the solubility of Kraft lignin (Table S2); lignin solubility data in the tested DESs, HBAs, HBDs alone, and their aqueous solutions (Table S3) (PDF)
