# Supplementary material for: Role of Deep Eutectic Solvent Precursors as Hydrotropes: Unveiling Synergism/Antagonism for Enhanced Kraft Lignin Dissolution
Source: ACS Sustain Chem Eng. 2024 May 31;12(23):8930–40. doi: 10.1021/acssuschemeng.4c02529 (PMC11168089; doi:10.1021/acssuschemeng.4c02529)
Supplement: Supplementary file 1 — sc4c02529_si_001.pdf [file sc4c02529_si_001.pdf]

THE ROLE OF DES PRECURSORS AS HYDROTROPES: UNVEILING SYNERGISM/ANTAGONISM FOR ENHANCED KRAFT LIGNIN DISSOLUTION

Filipe H. B. Sosa[1], Dinis O. Abranches[1], André M. da Costa Lopes[1,2], Mariana C. da Costa[3], João A. P. Coutinho[1*].

[1]*CICECO, Aveiro Institute of Materials, Department of Chemistry, University of Aveiro, 3810-193 Aveiro, Portugal*

[2]*CECOLAB – Collaborative Laboratory Towards Circular Economy, R. Nossa Senhora da Conceição, Oliveira do Hospital, 3405-155, Portugal*

[3]*School of Chemical Engineering (FEQ), University of Campinas (UNICAMP), 13083-852, Campinas, São Paulo, Brazil.*

*Corresponding author. E-mail address: jcoutinho@ua.pt

Number of Pages: 6

Number of Tables: 3

Number of Figures: 2

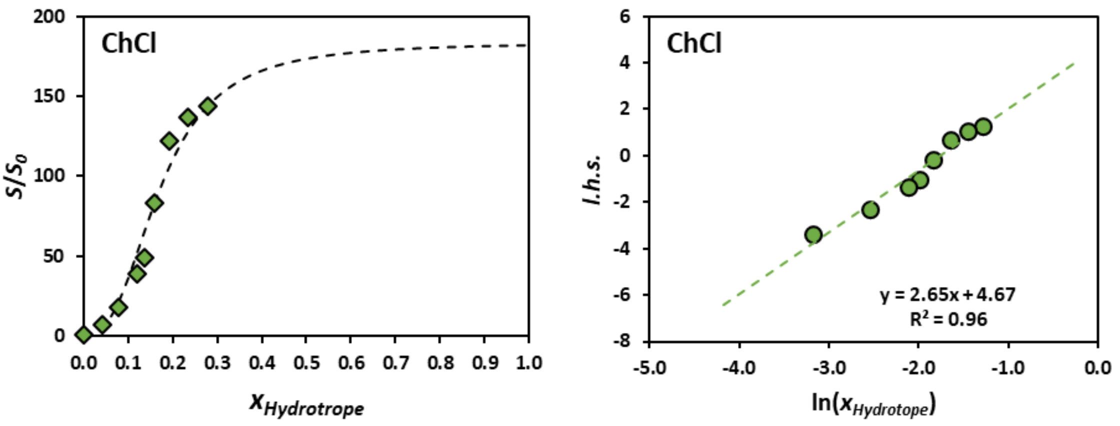

Figure S1. Left: solubility increase (S/S_0) of the Kraft Lignin in aqueous solutions of [ChCl] at 313.15 K (green diamond) along with the fitted curve of the cooperative hydrotropy model (- - -). Right: Experimental solubility data for Kraft lignin in aqueous solutions of [ChCl] at 313.15 K (green circles), in a linearized form (equation 1), where the y-axis represents the left-hand side (l.h.s) of equation 1 (- - -).

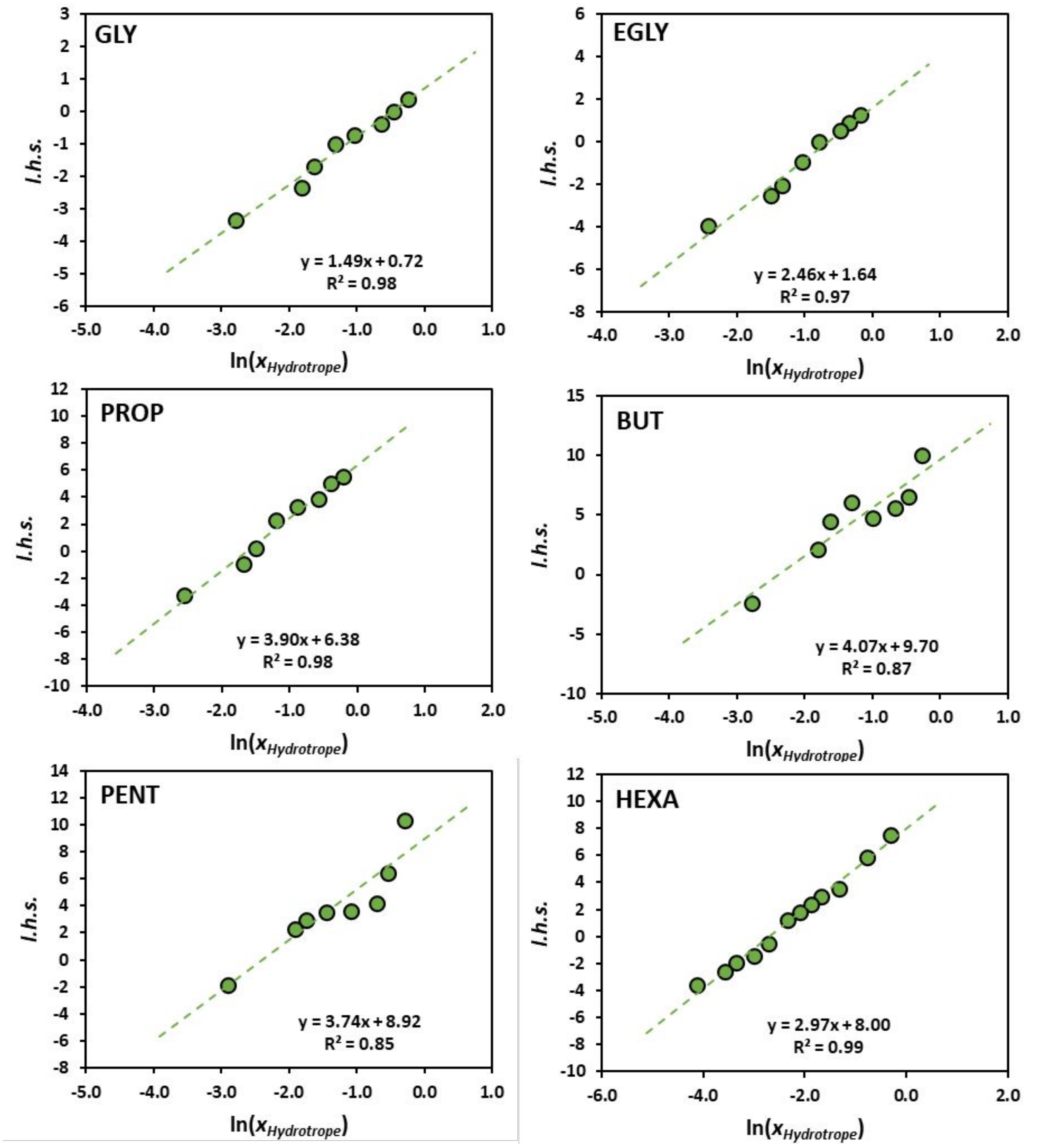

Figure S2. Experimental solubility data for Kraft lignin in aqueous solutions of GLY, EGLY, PROP, BUT, PENT, and HEXA at 313.15 K, in a linearized form (equation 1), where the y-axis represents the left-hand side (l.h.s) of equation 1 (- - -).

Table S1. Setschenow Constants (K_S) for lignin in solvents

System	K_S (kg/mol)
Choline Chloride	0.6444
Glycerol	0.1112
Ethylene glycol	0.1913
1,3-Propanediol	0.2976
1,4-Butanediol	0.4936
1,5-Pentanediol	0.5752
1,6-Hexanediol	1.6922
ChCl:GLY	0.2933
ChCl:EGLY	0.2978
ChCl:PROP	0.357
ChCl:BUT	0.6793
ChCl:PENT	0.9667
ChCl:HEXA	1.0344

Table S2. Parameters of the cooperative hydrotropy model for the solubility of Kraft lignin in alcohols, [ChCl] and HBD hydrotrope solutions

System	$(S/S_0)_{max}$	m	b
Choline Chloride	183	2.654	4.672
Glycerol	28	1.563	0.783
Ethanediol	288	2.459	1.640
1,3-Propanediol	166	3.904	6.377
1,4-Butanediol	176	4.068	9.696
1,5-Pentanediol	184	3.739	8.917
1,6-Hexanediol	204	2.970	7.997

Table S3. Solubility increase (S/S_0) of the Kraft Lignin in the studied aqueous solutions at 313.15 K.

x_{water}	S/S_0	x_{water}	S/S_0	x_{water}	S/S_0
**[Ch]Cl:GLY (1:2)**		**[Ch]Cl:PROP (1:2)**		**[Ch]Cl:BUT (1:2)**	
0.212	167.17±3.73	0.218	137.60±3.16	0.232	148.30±3.02
0.459	146.08±0.87	0.484	136.55±0.97	0.475	148.57±5.02
0.618	130.91±1.70	0.640	136.13±0.22	0.659	147.44±3.52
0.722	90.48±1.10	0.741	132.24±1.71	0.756	144.60±1.40
0.800	36.28±0.83	0.814	58.61±2.25	0.823	137.02±0.51
0.829	25.50±0.91	0.844	29.64±3.30	0.850	123.50±1.83
0.936	4.95±1.23	0.942	7.30±0.31	0.898	33.22±1.87
1.000	1.00±0.35	1.000	1.00±0.35	0.932	13.75±0.39
				0.947	9.95±0.21
				1.000	1.00±0.35
**[Ch]Cl:PENT (1:2)**		**[Ch]Cl:HEXA (1:2)**		**[Ch]Cl:HEXA (1:1)**	
0.255	156.31±2.70	0.272	170.03±2.98	0.262	141.95±0.01
0.520	153.84±0.1	0.543	168.39±0.90	0.537	141.24±2.69
0.671	153.69±3.26	0.697	168.33±5.76	0.686	142.62±1.62
0.772	142.91±0.62	0.785	168.55±0.10	0.779	142.56±2.94
0.835	136.72±0.22	0.845	168.61±3.21	0.842	141.36±4.25
0.861	128.56±0.32	0.869	166.37±1.89	0.869	98.04±0.96
0.905	67.25±1.84	0.910	100.68±2.08	0.908	67.23±2.37
0.937	32.57±0.38	0.940	31.16±0.94	0.951	18.88±0.10
0.950	18.24±0.39	0.953	19.99±0.2	1.000	1.00±0.35
1.000	1.00±0.35	1.000	1.00±0.35		
**[Ch]Cl:HEXA (1:3)**		**HEXA**		**[Ch]Cl**	
0.258	175.17±3.33	0.267	203.96±2.31	0.722	143.60±1.63
0.546	177.05±2.01	0.542	203.50±4.20	0.766	137.00±2.83
0.688	177.25±3.66	0.732	198.39±5.97	0.807	122.09±3.67
0.781	176.17±5.30	0.814	194.20±1.9	0.840	83.59±0.81
0.843	175.93±1.72	0.848	187.48±6.60	0.864	48.78±1.71
0.870	173.40±0.61	0.876	175.08±0.94	0.880	39.00±0.21
0.908	103.59±0.55	0.903	158.21±2.77	0.921	17.85±0.31
0.951	16.55±0.29	0.934	77.84±2.66	0.958	6.91±0.24
1.000	1.00±0.35	0.950	39.48±0.5	1.000	1.00±0.35
		0.965	26.14±0.26		
		0.972	15.26±0.31		
		0.984	6.64±1.26		
		1.000	1.00±0.35		

x_{water}	S/S$_0$	x_{water}	S/S$_0$	x_{water}	S/S$_0$
**GLY**		**EGLY**		**PROP**	
0.054	16.78±0.42	0.052	225.91±5.11	0.054	165.07±5.24
0.101	14.35±0.31	0.104	204.50±6.70	0.100	164.60±4.20
0.150	11.84±0.21	0.150	181.59±5.55	0.154	162.28±5.03
0.264	9.64±0.14	0.258	143.43±3.8	0.253	159.62±4.85
0.350	8.19±0.10	0.348	82.34±1.12	0.354	150.43±3.32
0.449	5.11±0.32	0.450	33.10±1.70	0.454	90.89±1.38
0.502	3.30±0.22	0.502	22.35±0.80	0.507	47.41±0.36
0.751	1.89±0.01	0.750	6.57±0.31	0.739	6.95±0.58
1.000	1.00±0.35	1.000	1.00±0.35	1.000	1.00±0.35
**BUT**		**PENT**			
0.057	175.54±6.04	0.056	184.44±5.79		
0.105	175.30±5.02	0.111	184.16±6.77		
0.157	174.93±5.99	0.150	181.76±5.56		
0.252	174.02±4.01	0.253	179.77±4.39		
0.349	175.16±5.89	0.360	179.16±6.04		
0.451	173.63±4.67	0.450	175.51±5.42		
0.505	157.00±3.68	0.497	167.53±4.99		
0.752	15.56±0.84	0.750	25.78±1.18		
1.000	1.00±0.35	1.000	1.00±0.35		
